# Haptic Error Modulation Outperforms Visual Error Amplification When Learning a Modified Gait Pattern

**DOI:** 10.3389/fnins.2019.00061

**Published:** 2019-02-19

**Authors:** Laura Marchal-Crespo, Panagiotis Tsangaridis, David Obwegeser, Serena Maggioni, Robert Riener

**Affiliations:** ^1^Gerontechnology and Rehabilitation Group, ARTORG Center for Biomedical Engineering Research, University of Bern, Bern, Switzerland; ^2^Sensory-Motor Systems (SMS) Lab, Institute of Robotics and Intelligent Systems (IRIS), Department of Health Sciences and Technology (D-HEST), ETH Zürich, Zurich, Switzerland; ^3^Reharobotics Group, Spinal Cord Injury Center, Balgrist University Hospital, Medical Faculty, University of Zurich, Zurich, Switzerland; ^4^Hocoma AG, Volketswil, Switzerland

**Keywords:** motor learning, motor adaptation, haptic guidance, error amplification, force disturbance, visual feedback, robotic gait-training, rehabilitation robotics

## Abstract

Robotic algorithms that augment movement errors have been proposed as promising training strategies to enhance motor learning and neurorehabilitation. However, most research effort has focused on rehabilitation of upper limbs, probably because large movement errors are especially dangerous during gait training, as they might result in stumbling and falling. Furthermore, systematic large movement errors might limit the participants’ motivation during training. In this study, we investigated the effect of training with novel error modulating strategies, which guarantee a safe training environment, on motivation and learning of a modified asymmetric gait pattern. Thirty healthy young participants walked in the exoskeletal robotic system Lokomat while performing a foot target-tracking task, which required an increased hip and knee flexion in the dominant leg. Learning the asymmetric gait pattern with three different strategies was evaluated: (i) No disturbance: no robot disturbance/guidance was applied, (ii) haptic error amplification: unsafe and discouraging large errors were limited with haptic guidance, while haptic error amplification enhanced awareness of small errors relevant for learning, and (iii) visual error amplification: visually observed errors were amplified in a virtual reality environment. We also evaluated whether increasing the movement variability during training by adding randomly varying haptic disturbances on top of the other training strategies further enhances learning. We analyzed participants’ motor performance and self-reported intrinsic motivation before, during and after training. We found that training with the novel haptic error amplification strategy did not hamper motor adaptation and enhanced transfer of the practiced asymmetric gait pattern to free walking. Training with visual error amplification, on the other hand, increased errors during training and hampered motor learning. Participants who trained with visual error amplification also reported a reduced perceived competence. Adding haptic disturbance increased the movement variability during training, but did not have a significant effect on motor adaptation, probably because training with haptic disturbance on top of visual and haptic error amplification decreased the participants’ feelings of competence. The proposed novel haptic error modulating controller that amplifies small task-relevant errors while limiting large errors outperformed visual error augmentation and might provide a promising framework to improve robotic gait training outcomes in neurological patients.

## Introduction

The interest in using robotic devices to provide more intensive and cost-effective gait training has increased during the last years (Marchal-Crespo and Reinkensmeyer, 2009). During robotic gait training, patients are physically assisted by a robotic device in order to move their legs into a physiological gait pattern (Marchal-Crespo and Riener, 2018). Robotic gait training has the potential to increase the training intensity while keeping patients in a safe and enjoyable environment (e.g., by using virtual reality) (Brütsch et al., 2010; Donati et al., 2016). However, robot-guided movements might, in some cases, decrease patients’ physical and mental effort during training (Israel et al., 2006). This could explain the limited functional gains observed after robotic gait training up to date (Dobkin and Duncan, 2012; Mehrholz et al., 2017).

It is generally accepted in the field of neurorehabilitation that recovery is a form of motor learning (Krakauer, 2006), and that understanding the underlying mechanisms during motor learning may facilitate the design of novel strategies to improve neurorehabilitation (Dietz and Ward, 2015). Active participation is thought to be an essential driving factor to elicit motor plasticity (Lotze et al., 2003; Behrman et al., 2006). Therefore, robotic rehabilitation could potentially hamper recovery if it promotes a decrease in cognitive and physical effort during training (Scheidt et al., 2000). “Challenge-based” controllers have been proposed in order to promote trainees’ participation. These challenging controllers, unlike guiding controllers that reduce errors during movement training, make motor tasks more difficult or challenging to perform (Marchal-Crespo and Reinkensmeyer, 2009).

Challenging controllers are based on the motor learning research that state that errors are fundamental signals to drive motor learning (Emken and Reinkensmeyer, 2005; Reisman et al., 2013). There is evidence that amplifying trajectory errors during walking using robotic forces accelerates the adaptive processes in healthy participants (Emken and Reinkensmeyer, 2005). Training with error amplification also enhanced learning of a complex locomotor task in initially more skilled healthy participants (Marchal-Crespo et al., 2017b). Error amplification during locomotion training resulted in more robust after-effects than assistive training (Yen et al., 2012). However, only few studies have tested for long-term retention (Helm and Reisman, 2015), and therefore, conclusions on the effect of error augmentation on motor learning of locomotor tasks should be taken cautiously. Furthermore, there are also studies that found that challenge-based controllers have a negative effect on participants’ motivation (Duarte and Reinkensmeyer, 2015), suggesting that error amplification might limit motor learning if it increases participants’ frustration during training.

Errors can also be visually augmented (i.e., the presented error on the display is distorted). In a relatively recent study, participants were asked to perform planar point-to-point reaching movements under a visuomotor rotation while holding the handle of a robotic device. Their arms were hidden by a screen showing them the reference trajectory as well as their current position, which was distorted in the experimental groups. The groups that had visual error amplification resulted in better learning outcomes than those who trained without augmented errors (Patton et al., 2013). Research in visual error amplification is quite recent. Exploration of visual error amplification has mainly focused on the upper limbs (Brewer et al., 2008; Celik et al., 2009; Patton et al., 2013; Basalp et al., 2016), although recent work has started to explore the possibility of using visual distortions on gait rehabilitation (Tobar et al., 2018). The use of visual error amplification is attractive, because it does not apply forces, and therefore, it does not create potential unsafe environments. Furthermore, it involves the use of virtual reality (VR), which has been shown to increase motivation and active participation during rehabilitation (Zimmerli et al., 2013; Bergmann et al., 2017). Including visual feedback (i.e., a VR representation of the desired and actual trajectory of the participants’ ankle), during training with a patient-cooperative minimally assistive robotic controller using the gait rehabilitation Lokomat (Hocoma AG, Switzerland), enhanced motor adaptation of a new gait pattern and resulted in improvements in locomotor function in stroke patients (Krishnan et al., 2012, 2013).

A recent study found that participants with more variable movements during baseline could more rapidly adapt to a perturbation and learn a new skill than participants with low movement variability (Wu et al., 2014). This is in line with recent research that states that during the first stages of learning, error exploration (i.e., the active exploration of new motor tasks) is crucial to boost motor learning (Huberdeau et al., 2015). Therefore, increasing movement variability during training might result in better motor learning. A possible approach to increase movement variability is to apply randomly varying feedforward forces (i.e., haptic disturbance) during training (Rüdt et al., 2016). In a motor learning study on upper limbs, adding haptic disturbance while training a tracking task resulted in better tracking skills than training with haptic error augmentation and training without disturbances (Lee and Choi, 2010). We recently found that adding random haptic disturbance during training a locomotion task increased muscle activation and seemed to enhance attention and motor learning (Marchal-Crespo et al., 2014a,b, 2017b).

Haptic guidance seems to be particularly helpful for initially less skilled participants (Marchal-Crespo et al., 2010), while error amplification was found to be more beneficial for more skilled participants (Cesqui et al., 2008; Milot et al., 2010). This is in line with the challenge point theory, which states that optimal learning is achieved when the difficulty of the task is appropriate for the participant’s level of expertise (Guadagnoli and Lee, 2004). Therefore, matching the robotic training strategy to the trainee’s skill level may provide the greatest opportunity for learning (Metzger et al., 2014).

Motivation has been suggested to play a key role during motor learning and neurorehabilitation (Reinkensmeyer and Housman, 2007; Novak et al., 2014). Several studies have shown that increasing participants’ perceived competence and intrinsic motivation during training can enhance the acquisition of new motor skills (Ávila et al., 2012; Saemi et al., 2012; Widmer et al., 2016). Motivation may, in some training situations –e.g., when it is associated with high reward– improve learning consolidation (Trempe et al., 2012). The close relationship between task difficulty and motivation has been extensively studied since the early 20-th century [e.g., *difficulty law of motivation* (Ach, 1935)]. According to the Flow theory and Self-determination theory, the maximum intrinsic motivation is achieved when the difficulty of the task optimally challenges the participant. It has been suggested that the relationship between perceived task difficulty and motivation follows an inverted U-shaped function (Ma et al., 2017). The optimal level (i.e., the apex of the curve, named *flow*) is described as “*an intrinsically motivating and fully engaging state of consciousness”* (Csikszentmihalyi, 2014). Interestingly, recent studies have shown that participants report higher levels of intrinsic motivation when they slightly underperform in the tasks (the so-called ‘close missing’), compared to performing perfectly (*boredom channel*) or far worse than required (*anxiety channel*) (Abuhamdeh et al., 2015; Ma et al., 2017). Therefore, although reward might enhance motor skill learning, performing systematically well might decrease motivation, compared to closely missing the target.

An important concept in error-based motor learning theory is the idea that participants must explore the task by themselves (exploration) and exploit current reliable knowledge (exploitation). These two processes are often considered to be antagonistic (the so-called ‘exploration-exploitation tradeoff’). However, this tradeoff might be bypassed using robotic devices. An optimal framework for motor learning might consist in limiting unsafe and frustrating large errors, which might result in participants stumbling or falling, by using robotic haptic guidance (i.e., favoring exploitation), while augmenting movement variability and awareness of small learning-relevant errors by using error amplification and random haptic disturbance (i.e., enforcing exploration). This optimal framework might influence not only the exploration and exploitation processes that have a direct effect on motor learning, but might also increase motivation. Bringing participants to practice in an area close to the flow apex (in the close missing area) might increase participants’ motivation and enjoyment.

In this experiment, we investigated the effect of training with novel visual and haptic error modulating strategies on motivation and learning of a modified gait pattern. Learning with three different error modulating strategies was evaluated: (i) no disturbance: no robot disturbance/guidance was applied, (ii) haptic error amplification: unsafe and frustrating large errors were limited with haptic guidance, while haptic error amplification enhanced awareness of task-relevant errors, and (iii) visual error amplification: visually perceived errors were amplified in a virtual reality environment. We also evaluated whether increasing the movement variability during training by adding randomly varying haptic disturbances on top of the other training strategies further enhanced learning. Thirty healthy young participants walked in the robotic gait trainer Lokomat while performing an ankle target-tracking task which required an increased hip and knee flexion in the dominant leg. We analyzed participants’ motor performance and self-reported intrinsic motivation before, during and after training. We hypothesized that training with error amplification, either visually or haptically, would result in better motor learning than training without error amplification. We hypothesized that limiting large errors during haptic error amplification would increase self-perceived competence, enjoyment and, therefore, motivation. Finally, we expected that adding haptic disturbance on top of the other training strategies would increase movement variability during training and would further enhance motor learning.

## Materials and Methods

### Lokomat

**FIGURE 1:**
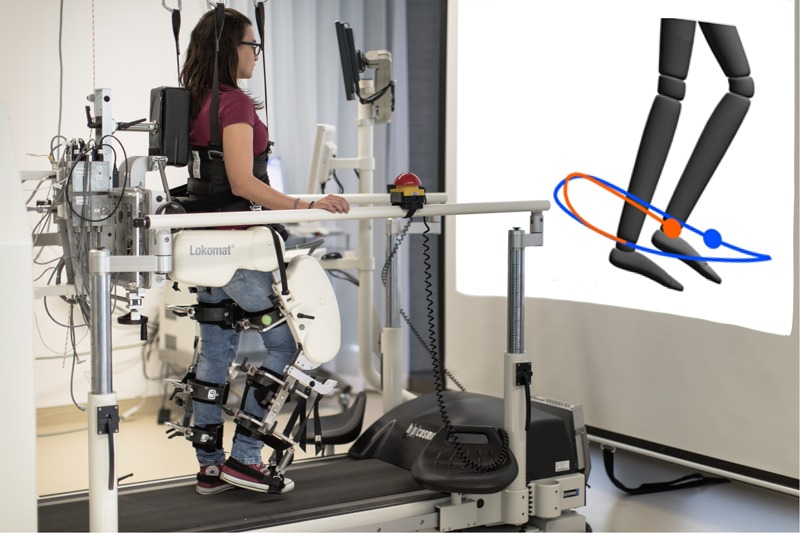
The Lokomat^®^ (Hocoma AG, Switzerland) is a bilateral gait robotic orthosis that, together with a body-weight support system and a treadmill, controls patient’s hips and knees movements in the sagittal plane (Tsangaridis et al., 2018). The participant in this figure consented to the publication of her image.

Even though the error modulating controllers presented in this paper are applicable to different robotic gait-training systems, the presented experiment was performed with the Lokomat^®^ (Hocoma AG, Switzerland). The Lokomat is a commercial available robotic gait trainer that consist of two leg orthoses, a body weight support system and a treadmill (Figure 1) (Riener et al., 2010). Each orthosis can induce flexion and extension movements in the hip and knee joints in the sagittal plane through linear drives. Ankle dorsiflexion during the swing phase can be supported through passive foot lifters. Each leg orthosis is fixed to a frame that allows for passive vertical translations and keeps the orientation of the pelvis segment constant. A sophisticated combination of passive elastic and active dynamic systems, the “Lokolift,” allows for a constant unloading of patient’s weight during treadmill walking (Frey et al., 2006).

### Experimental Task

**FIGURE 2:**
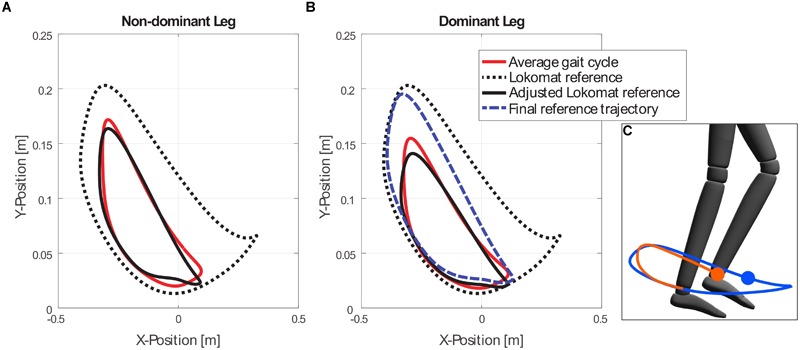
Example of the ankle trajectories that resulted from applying forward kinematic analysis to a participant’s average hip and knee joints (θ_ave_), the Lokomat original (θ_Lok_) and adjusted Lokomat references (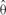
_Lok_), and the final reference trajectory (θ_ref_) in the non-dominant **(A)**, and dominant legs **(B)**. The final trajectory is the result of increasing the hip and knee angle ROM of the dominant leg by 20%. The final reference ankle trajectory was shown on the screen **(C)** together with the actual ankle position and an avatar representation of the legs (with dominant leg on top).

The experimental task consisted in tracking with the dominant ankle a desired trajectory presented on a visual display, while the non-dominant leg was fully guided by the robot. Participants’ legs were visually displayed as an avatar on a large screen in front of them (Figure 1). Participants were requested to track a blue dot (reference position), which moved along the reference trajectory, with the ankle of their dominant leg. In order to facilitate the task, an orange dot indicated the participant’s dominant ankle actual position, and an orange trace showed the path followed by the ankle for a certain time (Figure 2C).

The target trajectory corresponded to a gait pattern that required a 20% increase in the dominant leg’s average hip and knee joint angular range of motion (ROM). A similar gait pattern was successfully employed in previous experiments performed with the Lokomat (Krishnan et al., 2013; Ranganathan et al., 2016). The individual average hip and knee angles across the gait cycle for both legs for each participant (θ_ave_) were calculated after a 2 min calibration test, where participants freely walk in the Lokomat without any haptic guidance/disturbance. The recorded time series were then split into single steps. The start of every step was defined as the point when the left hip joint angle reached a maximum. The default joint references loaded in the Lokomat (θ_Lok_) are based on pre-recorded standard joint trajectories (Colombo et al., 2000). These trajectories can be manually modified to better fit the participant’s particular gait pattern by changing the *Gain* and *Offset* parameters. We developed an algorithm that calculates the *Gain* and *Offset* parameters that better fit the pre-recorded Lokomat joint references to each participant’ specific gait pattern.

The *Gain* is calculated for each joint and leg independently by dividing the ROM (i.e., the difference between the maximum and minimum joint angles) of the measured average joint trajectory (*ROM_ave_*) over the ROM of the Lokomat reference joint trajectory (*ROM_Lok_*).

(1) $$Gain=\frac{ROM_{\mathrm{ave}}}{ROM_{\mathrm{Lok}}}$$

The *Offset* was calculated by computing the average deviation between the average joint trajectories (θ_ave_) and the Lokomat default trajectory (θ_Lok_) multiplied by the *Gain* (calculated with Eq. 1).

(2) $$Offset=\frac{\sum_{i=1}^{m}\left(\theta_{\mathrm{ave},i}-\theta_{\mathrm{Lok},i}•\mathrm{Gain}\right)}{m}$$

where *m* is the number of data points in each gait cycle (m = 250). The fitted Lokomat reference joint trajectories for each joint (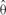
_Lok_) are then calculated as:

(3) $${\hat{\theta }}_{\mathrm{Lok}}=\left(Gain•\;\theta_{\mathrm{Lok}}+Offset\right)$$

We computed the final desired joint trajectories by increasing the gain of the fitted Lokomat reference 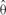
_Lok_ by 20% in the dominant leg. The desired and actual ankle trajectories presented on the visual display were then calculated by employing forward kinematics analysis of the hip and knee joint angles (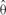
_Lok/hip_, 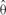
_Lok/knee_), and the measured segment lengths of the thighs and shanks (l_thigh_, l_shank_) for each participant (Figure 2).

### Training Strategies

We developed new training strategies to haptically or visually modulate movement errors in order to enhance motivation and learning of a modified gait pattern. The design and evaluation of the haptic disturbance and error modulating haptic controllers for the Lokomat were described in detail in Rüdt et al. (2016). Here, only a brief summary is given for completeness. Similar haptic disturbance (Marchal-Crespo et al., 2017a) and error modulating controllers (Marchal-Crespo et al., 2017c) were developed to perform motor learning experiments with ARMin, a 7 degree-of-freedom (DoF) robotic exoskeleton for upper limb rehabilitation. The haptic controllers employed in the present experiment, however, were developed in joint coordinates and were not based on the end-effector trajectories. The visual error amplification in joint coordinates was newly developed for the current experiment.

#### No Disturbance

In no-disturbance mode, the robotic device does not help nor disturb the participants during walking. The robot works with zero-torque control, in such a way that the interaction torques between robot and human are minimized by letting the robot follow the participant’s self-selected movements (Riener et al., 2005). Friction and gravity are compensated to improve the transparency of the orthoses.

#### Error Modulating Haptic Controller

In order to haptically augment errors, we developed a controller that provides amplifying torques (T_amp_) that direct the joint angles away from the desired position (Rüdt et al., 2016). These amplifying torques are calculated using a proportional controller in joint space of the form:

(4) $$T_{\mathrm{amp}}=\lambda_{\mathrm{amp}}•\left(\theta_{act}-\theta_{\mathrm{ref}}\right)=\lambda_{\mathrm{amp}}•e$$

where θ_ref_ is the reference joint angle, and θ_act_ is the measured angle (θ_hip_ or θ_knee_). With this formula, the error amplification torques would increase proportionally to the tracking errors. However, participants are able to apply only a certain maximum torque to correct their movements. Therefore, come back to the desired joint position would be challenging if the error is especially large. In order to limit the amount of torque amplification and limit large errors that can be unsafe and frustrating for the participants, we realize a conversion toward haptic guidance when the error is larger than a predefined allowed error (e_turn_). In this way, the system amplifies small errors (e < e_turn_) but prevents participants from performing large errors (e > e_turn_). This is achieved by making the proportional gain λ_amp_ a function of the participants’ ongoing error (Rüdt et al., 2016):

(5) $$\lambda_{\mathrm{amp}}=\lambda_{\max}•\left(\frac{2}{1+exp\left(k•\left(\left|e\right|-e_{\mathrm{turn}}\right)\right)}-1\right)$$

**FIGURE 3:**
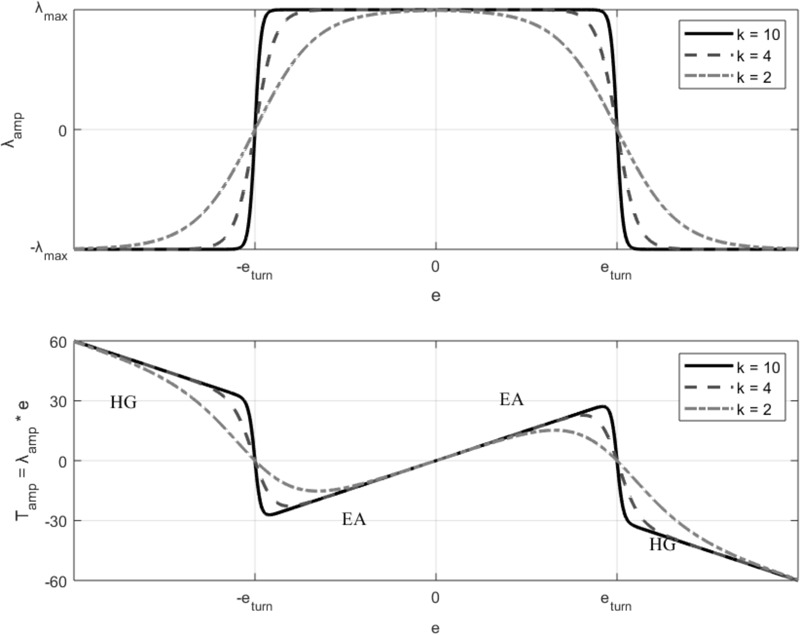
**(Up)** The impedance gain in the error modulating haptic controller (λ) depends on the participants’ ongoing error following a combined sigmoid function. **(Bottom)** The resulting torque (T_amp_) amplifies small errors (e < e_turn_) with error amplification (EA), but prevents participants from performing large errors (e > e_turn_) with haptic guidance (HG).

The impedance gain follows the superposition of two sigmoid functions (Figure 3, up). The gain is maximal (λ) when the error (*e*) is equal to zero. The controller applies error amplification as far as the error is within the allowed error (e_turn_) (Figure 3, bottom). For larger errors, participants are directed back to the desired position with haptic guidance. The impedance gain saturates for big errors (–λ_max_). The error range in which the impedance gain remains constant, and the transition between error amplification and haptic guidance around e_turn_ can be tuned using the parameter *k*. Small values of *k* result in slow soft transitions between control modes but reduce the width of the impedance gain saturation area (Figure 3, up). In order to provide a relative soft transition between controllers, while keeping an impedance gain saturation area relatively large, the slope *k* of the sigmoid was set to 10. In order to allow a certain tracking accuracy without excessive participants’ physical effort, a small λ gain of 25 Nm/° for the hip and 5 Nm/° for the knee were selected. The predefined allowed error (e_turn_) was fixed to 8°. Participants’ legs are attached to the Lokomat through fabric cuffs fastened with Velcro, and therefore, small relative movement between the participants’ and robot links can be minimized, but not totally canceled (i.e., keeping the error exactly at zero is hardly possible). In order to avoid amplifying errors that are not directly related to the participants’ own performance, errors smaller than a threshold (2°) are not amplified. The error amplification torque is then multiplied by a sigmoid function derived from the safety constraints described in Section “Constraints,” and input to a close-loop torque controller.

#### Visual Error Augmentation

The visual error augmentation algorithm displays the participant’s actual ankle position farther away from the reference point than it actually is. The visual error amplification algorithm works in joint coordinates to resemble the haptic error amplification algorithm. The hip and knee joints represented by the VR avatar when visual error amplification is applied (θ_shown, hip_, θ_shown, knee_), are calculated as follows:

(6) $$\left[\begin{array}{c} \theta_{shown,\;hip}\\ \theta_{shown,\;knee} \end{array}\right]=\left[\begin{array}{c} \theta_{act,\;hip}\\ \theta_{act,\;knee} \end{array}\right]+\left[\begin{array}{c} \theta_{act,\;hip}-\theta_{\mathrm{ref},\;\mathrm{hip}}\\ \theta_{act,\;knee}-\theta_{\mathrm{ref},\;\mathrm{knee}} \end{array}\right]•\left(\alpha_{\mathrm{amp}}\right)$$

The visual error amplification gain (α_amp_) was set to 0.2 (i.e., 20% amplification). This value was selected based on previous experiments that showed that gains bigger than 40% result in participants’ confusion when tracking a continuous repetitive trajectory (Basalp et al., 2016). With these new calculated joint angles, forward kinematic analysis was employed to compute the position of the ankle shown on the VR screen.

We added some saturation constraints on the visual amplification algorithm in order to keep the VR avatar realistic. In an obvious case, we prevented the knees from going into hyperextension (θ_shown, knee_ ≥ 0°). We also saturated the amount of amplified error. In preliminary tests, we observed that an added 15° error was the upper limit where a participant could still believe that the movement shown on the VR was his or her own movement.

#### Haptic Disturbance

The idea of the random haptic disturbance algorithm is to increase the participants’ error variability while training with their main training strategy (i.e., no disturbance, haptic or visual error amplification). By increasing the variability, we seek to push the participants away from their “comfort zone,” encouraging the exploration of the task environment (Rüdt et al., 2016).

The haptic disturbance controller generates torque pulses that last for 0.15 s. The occurrence of the pulses is pseudorandomized. Every 0.1 s, a random number is drawn from a standard uniform distribution. If this value is smaller than 0.2, a disturbance is applied. This results in an average pulse frequency of 2 Hz. However, the algorithm also enforces a minimum idle time between pulses of 0.1 s and a maximum of 0.5 s, and therefore, the timing of the pulses cannot be considered totally random. The magnitude of the disturbance torques are drawn from a uniform distribution in the range from -1 to 1. This magnitude is then multiplied by the maximum possible disturbing torque (10 Nm). The Lokomat safety control shuts down the system when a sudden change in the joint angles is detected. Therefore, a rate limiter was implemented to guarantee that the disturbance torque takes 0.05 s to reach its full value, maintains it for 0.05 s, and returns to zero in 0.05 s. The disturbance torques are then multiplied by a sigmoid function –derived from the safety constrains described in next section– and applied simultaneously to the hip and knee joints of the dominant leg on top of the other training strategies.

#### Constraints

The goal of the novel haptic error modulating training strategies is to increase movement variability and kinematic errors in a motivating and safe way. In order to achieve a safe environment for gait training, different constraints were introduced that prevented participants from stumbling (Rüdt et al., 2016).

##### Constraint 1: no application during stance phase

The stance phase starts when the heel strikes the ground and last until the toe leaves the ground (i.e., determines the time when the foot is in contact with the ground). The application of haptic error amplification and haptic disturbance during stance might make the leg buckle and result in stumbling. Therefore, the use of these disturbing torques is limited to the swing phase. A swing phase detection algorithm that uses the online measurements of both legs’ hip (θ_hip_) and knee (θ_knee_) angles, and the lengths of each participant’s thighs (l_thigh_) and shanks (l_shank_) was developed (see Rüdt et al., 2016 for more detailed information). This algorithm calculates the vertical distance from the hip joint to the heel (y_heel_) and the toe (y_toe_) for each leg using forward kinematics.

The maximum y_heel_ and y_toe_distances for each leg are selected and compared between legs (y_diff_). If their difference y_diff_ is greater than an ad hoc selected threshold (y_threshold_ = 0.02 m), the leg with the smallest distance is considered to be in swing phase.

##### Constraint 2: continuous transition

It is important to guarantee a smooth transition between the application of the haptic error amplification and disturbing torques during swing phase and the disturbance-free stance phase enforced by the first constraint. In order to apply a soft transition between swing and stance phases, we calculate a sigmoid function that changes its value from one to zero depending on the difference between the two legs’ vertical distance (y_diff_) (Rüdt et al., 2016). The disturbance torques are then multiplied by this sigmoid function in order to limit their magnitude in the swing-stance and stance-swing transitions. In order to preserve participants’ safety during training, the constraints are only applied to the haptic error amplification and disturbing torques, but have no effect on the haptic guidance. Haptic guidance would always be applied during the stance phase, if the error is larger than the predefined allowed error (e_turn_).

### Experimental Protocol

**FIGURE 4:**
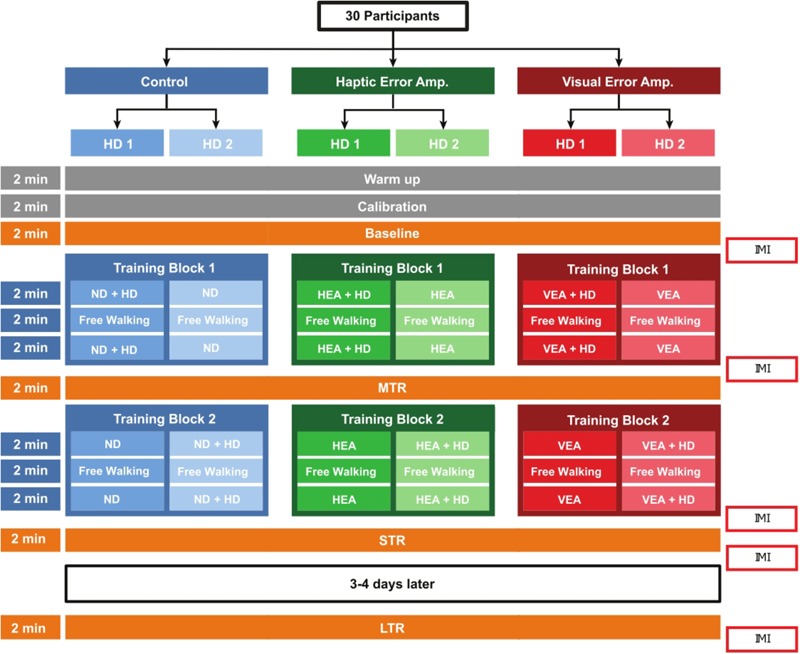
Experimental protocol. Participants were randomly allocated to one of three training groups (Parallel design: no disturbance [ND/Control], haptic error amplification [HEA], and visual error amplification [VEA]). Within these groups, participants were split into two groups (cross-over design), depending whether they started training with haptic disturbance (HD) added on top of their main training strategy (HD1), or they started training without haptic disturbance (HD2). Motor learning was evaluated at mid-training retention (MTR), short-term retention (STR), and long-term retention (LTR). After baseline, after the first and second training blocks, and after the short- and long-term retention tests, participants responded to six statements (Table 1) selected from the Intrinsic Motivation Inventory (IMI).

The study was approved by the ethical committee of ETH Zürich and conducted in compliance with the Declaration of Helsinki. Thirty young healthy participants (15 females), 26.0 ± 2.8 years old, gave written informed consent to participate in the study. All participants, except for one, were right footed, as determined by their preferred leg to kick a ball as far as possible (Krishnan and Williams, 2009). Participants were randomly allocated to one of three training groups (Parallel design: no disturbance [Control], haptic error amplification [HEA], and visual error amplification [VEA]). Within these groups, participants were again split into two groups (cross-over design), depending whether they started training with haptic disturbance (HD) added on top of their main training strategy (HD1), or on the contrary, they started training without haptic disturbance (HD2). An overview of the study protocol is depicted in Figure 4. Participants were not informed in which group they were allocated but were informed about the possibility that the robot could help or disturb them while executing the task.

Participants were positioned in the Lokomat using the usual Velcro cuffs at the pelvis, thighs and shanks and the length of each robot segment was adjusted to correctly align the hip and knee joints of the exoskeleton with the participants’ joints. We employed passive foot lifters to support ankle dorsiflexion during the swing phase. The experiment was performed with a treadmill speed of 1.5 km/h at a pace of around 57 steps per minute and with 30% body weight support, provided through a harness. A relative low speed was selected to ensure that the task was not too challenging for the participants, and to allow them to correct the errors through the step cycle.

The experiment consisted of two experimental days which were 3–4 days apart. On day 1, participants started to freely walk in the Lokomat for 2 min to get used to the robot in no disturbance mode (warm-up). Participants were then requested to freely walk for another 2 min (calibration) in order to determine the ankle target template (see section “Experimental Task”). Participants were verbally instructed to walk as naturally as possible. Once the target trajectory for each joint and each leg was computed, we turned on the VR game and participants were informed that the experiment would start. During baseline (2 min), participants were instructed to follow a blue dot that moved on the target ankle trajectory presented on the screen in front of them, with their dominant leg (signaled with an orange dot) whenever the visual feedback was available. Their non-dominant leg was fully guided by the robot, while they could freely move their dominant leg.

After a short break (<1 min), participants started the first training block. A training block consisted of: 2 min of training (training 1.1), 2 min of free walking (FW1), and again 2 min of training (training 1.2). The VR game and the participant’s specific training controller (Control, HEA or VEA in the dominant leg and full guidance on the non-dominant leg), were active during training, and turned off during free walking. Participants were verbally instructed to walk naturally during the free walking test. Participants performed two training blocks of 6 min each. Half of the participants (HD1) trained with haptic disturbance on top of their specific training strategy during the first training block. Between the first and second training blocks, a 2-min mid-training retention (MTR) test took place. During the second training block (training 2.1, FW2, and training 2.2), participants in the HD2 group trained with haptic disturbance on top of their specific training controller, while participants in HD1 trained without haptic disturbance. After the second training block and a 5-min break, a short-term retention (STR) test was performed. The total experimental time was around 1 h. Participants were invited to return after 3–4 days to perform the long-term retention test (LTR). All the retention tests followed the same structure as baseline.

In total, participants performed the tracking task (with or without error augmentation strategies) during seven time intervals of 2 min each in the first experimental day. This number was selected based on the limited previous experimental results that showed significant performance improvements in a very similar task after performing the tracking task during four training intervals of 2 min each (Krishnan et al., 2013). Performance in the tracking task saturated after performing the task for more than five training intervals of 2 min each (Ranganathan et al., 2016). Although the previous experimental results assessed performance during training, rather than learning, we hypothesized that the task was relatively easy and could be mastered in a short time (Marchal-Crespo et al., 2010; Winstein et al., 1994).

We assessed participants’ subjective experience with the experimental task after baseline, after the first and second training blocks, and after the short and long retention tests (Figure 4). We employed six statements (Table 1) from the well-established Intrinsic Motivation Inventory (IMI, Ryan, 1982). The IMI has been successfully employed in several motor learning experiments to assess intrinsic motivation (Duarte and Reinkensmeyer, 2015; Marchal-Crespo et al., 2017c). The full questionnaire assesses seven motivational subscales (with a total of 45 questions). In the present study, we focused in assessing interest/enjoyment, perceived competence, and effort/importance. Participants ranked their agreement with each of the six statements using a Likert scale between 1 and 7 points; 1 indicated “I disagree completely” and 7 indicated “I agree completely.” The questions were presented in German and English. Answers from the same questions at different experimental times were always visible.

**Table 1:** IMI Questionnaire.

IMI questions	Subscale
(1) This exercise of following the blue point was fun to do.	Interest/enjoyment
(2) After working at this exercise of following the blue point for a while, I felt pretty competent.	Perceived competence
(3) I tried very hard to follow the blue point.	Effort/importance
(4) It was important to me to do well following the blue point.	Effort/importance
(5) I am satisfied with my performance at following the blue point.	Perceived competence
(6) I would describe this exercise following the blue point as very interesting.	Interest/enjoyment

*The six statements selected from the Intrinsic Motivation Inventory in order to assess participants’ interest/enjoyment, perceived competence, and effort/importance (IMI, Ryan, 1982)*.

### Data Processing

The knee and hip angles were recorded by the robot at 50 Hz. All data was processed with Matlab (MathWorks, Natick, MA, United States). The recorded angles were smoothed using a moving average filter with a span of five. The actual and reference ankle positions were determined using forward kinematics analysis of the hip and knee joint angles along with segment lengths of the thigh and shank measured for each participant. For the analysis, the time series collected from each participant were segmented into single steps using a heel strike detection algorithm (Bernhardt et al., 2005). The ankle trajectories were then normalized to 250 discrete points via interpolation in order to have equal number of time frames for each gait cycle. In average, during free walking, the first time frame would correspond to heel strike (start of stance phase), the pre-swing phase would start around the time frame 100 (40% of the gait cycle) and swing phase would start around the time frame 150 (60% of gait cycle) (see Supplementary Figure A1). In order to avoid transitory effects (i.e., participants needed time to synchronize their gait with the robot), the first five steps recorded for each participant during a training or retention test were removed.

Different variables were extracted from the ankle position to evaluate the participants’ spatio-temporal performance (error) and movement consistency (variability). The tracking error (*e*_i,t_) in each time frame (*t*) of a gait cycle (*i*) was obtained by calculating the absolute distance between the actual and the desired ankle position at each specific time frame (250 discrete points per gait cycle). The average trajectory tracking error is then calculated by averaging the tracking error in each time frame over all gait cycles. The experimental task consisted in tracking a desired position over time, therefore, the tracking error includes timing and spatial mismatches between desired and actual positions.

We also evaluated the spatial errors using dynamic time warping (DTW) with the weighting of the temporal shift set to zero (Giese and Poggio, 2000). The spatial error provides important information regarding how close was the performed ankle trajectory to the desired ankle reference trajectory. This information is valuable to assess whether participants learned to perform the asymmetric desired trajectory, contrary to the tracking error, which is employed to assess how precise were the participants in tracking the desired ankle movement at each time frame. The spatial error in each time frame of the gait cycle was obtained using the MATLAB built-in function *dtw*. During DTW, the total distance between the two temporal sequences is computed as the minimum sum of the Euclidean distances between the column vectors of these sequences. The reader is referred to (Giese and Poggio, 2000) for a detailed description of the DTW algorithm. In our case, column vectors represent the time frame during the gait cycle (a total of 250 time frames, where the first value corresponds to heel strike), while row vectors are the Cartesian coordinates (*x* and *y*) of the ankle’s position. Hence, we extracted the spatial error at each time frame (*t*) of a gait cycle (*i*) by comparing the measured trajectory of each step with the corresponding reference trajectory.

In order to investigate whether adding haptic disturbance increased the movement variability, we also calculated the variability of the spatial error. The variability is defined as the average trajectory spatial error from each step to each other step. The trajectory spatial error of each step to each other step is calculated using the *dtw* MATLAB function, creating an *n* × *n* symmetric matrix. The trajectory variability is then obtained by calculating the average trajectory of one half of the matrix.

In order to evaluate whether training the asymmetric gait pattern modified the gait pattern during free walking tests (transfer), we calculated the asymmetry between trained and untrained legs for the hip and knee joints during calibration and free walking blocks (FW1 and FW2). The asymmetry performance metric was defined as the percentage difference between the ROM of the trained and untrained leg joints. We calculated the asymmetry for the hip and knee joints independently.

(7) $$Asymmetry_{hip/knee}=\frac{ROM_{trained\;hip/knee}-ROM_{untrained\;hip/knee}}{ROM_{untrained\;hip/knee}}$$

Positive asymmetry values imply bigger ROM in the trained joints, while big divergence from zero indicates high asymmetry between the legs. The asymmetry variable is a discrete value calculated for each single step. In order to evaluate differences between trained and non-trained joints within a continuous gait cycle, we calculated the difference between the joint trajectories of the trained and the untrained legs (θ_trained hip/knee_ - θ_untrained hip/knee_) within each gait circle.

### Statistical Analysis

We excluded one outlier from the analysis (from the HEA group). We detected one participants who performed systematically worse than the others [his/her performance variables systematically lied out of the 1.5 inter quartile range (IQR) in most test and training blocks]. We noted that the calibration process in this particular participant resulted in exceptional large joint ROMs. This probably led to a target trajectory which was too challenging to reach, as the target trajectory is proportional to the ROMs calculated during calibration (a 20% increase in the ROM). After the exclusion of the outlier, the normality of the data was confirmed using Kolmogorov–Smirnov tests.

We used linear mixed effects (LME) analysis to evaluate the effect of the different training groups (Control, HEA, and VEA), time (e.g., baseline, mid-training retention, short-term retention) and HD factor (addition of haptic disturbance in the cross-over design) on the performance variables. We employed the absolute mean values of each performance variable during a gait cycle as dependent variables (i.e., for each gait cycle we took the mean of the 250 time frames). We used the *lme4* package (Bates et al., 2015) for *R* (R Core Team, 2017)). Initially, we entered as fixed effects the interaction between training groups, time and HD factor into the LME model, while participants were modeled as a random factor to account for the by-subject variation. With backward elimination of the non-significant fixed effects using model comparison analysis with the Akaike Information Criterion (AIC), the HD factor was eliminated from the original model, since the addition of haptic disturbance did not have any significant effect on any of the error performance variables. Therefore, the final model employed to fit our data had the form:

(8) $$Performance\;variable\;\sim \;group^{*}time+\left(1|subject\right)+\varepsilon$$

The *lmerTest* package in *R* (Kuznetsova et al., 2017) was used to test significance of the effects while it provides degrees of freedom and *p*-values for the *t* and type III F-tests with Satterthwaite degrees of freedom approximation. We report the estimates (β), standard errors (*SE*), confident interval (CI) with parametric bootstrapping, and significant levels (*p*).

Participants were repositioned in the Lokomat when they returned for the second experimental day. Although the examiner employed the same individual participant-based parameters in both days (e.g., length of segments in the orthoses, cadence, etc.), it is challenging to precisely reposition participants in an exoskeleton (Marchal-Crespo and Riener, 2018). Different alignments between the anatomical human and robotic joint axes within the two experimental days may have an impact in the end-effector kinematics (Bartenbach et al., 2015) and introduce variability into the data that could potentially influence the power of our statistical model. Thus, the performance in long-term retention compared to baseline and short-term retention was separately investigated using two independent two-level LME models (baseline-LTR and STR-LTR). We employed ANOVAs in order to test whether the performance variables during baseline were different between training groups. *Post hoc* comparisons were performed with Tukey corrections. In order to investigate whether the performance of the participants trained with HEA significantly changed between training and retention blocks (i.e., whether participants relied on the provided torques during training), we compared the performance between training 1.2 and mid-training retention and between training 2.2 and short-term retention with paired *t*-tests. We investigated the effect of adding haptic disturbance on the variability of the spatial error during training using linear mixed effects models with the interaction of training groups and haptic disturbance groups as the fixed effects.

For safety reasons, the training controllers are only active during swing phase. Therefore, no difference between training groups were expected during the stance phase. In order to get a better insight into participants’ performance during the continuous gait cycle, we also performed statistical analysis on the continuous performance variables using Statistical Parametric Mapping (SPM). SPM is suitable for the analysis of smooth continuum changes in biomechanical data and allows for topological analysis of the data. The SPM main advantage over the mean performance variable approach, is that statistical results are presented directly in the original sampling space without any need for data reduction and discretization of the dependent variables (Friston et al., 2007). Therefore, the differences between/within training groups can be localized within a gait cycle (i.e., vectors with 250 time frames). The SPM analysis was performed using the open-source *spm1d* package (Pataky, 2012) in Python (Python Software Foundation, version 2.7^1^). Two-way ANOVAs with repeated measures on the time factor (baseline – training 1.1 – training 1.2 – training 2.1 – training 2.2 when analyzing training performance, and baseline – mid-training retention – short-term retention when analyzing motor adaptation), and main effect of training group (Control, HEA, and VEA) were performed with the continuous tracking error as dependent variables. A two-way ANOVA with repeated measures on the time factor (calibration – free walking 1 – free walking 2), and main effect of training group (Control, HEA, and VEA) was performed with the continuous difference between dominant and non-dominant knee trajectories. One-way ANOVAs were used in further comparisons if the two-way ANOVA was significant.

We evaluated the effects that the different training strategies had on the three IMI subscales: interest/enjoyment (Q1, Q6), perceived competence (Q2, Q5), and effort/importance (Q3, Q4). We used non-parametric independent samples Kruskal–Wallis tests in order to evaluate potential differences between training groups in the responses to each IMI subscales after baseline. We compared the responses to each IMI subscales after the first and second training blocks, and after the short- and long-retention tests relative to the responses after baseline using Kruskal–Wallis test with training group as the main factor. If the Kruskal–Wallis test was significant, Mann–Whitney Test range was used to perform pairwise comparisons. A Mann–Whitney Test was also employed to test the effect of adding haptic disturbance on the changes of each IMI subscale scores from baseline to first training block, and from first to second training blocks.

Statistical analysis of the IMI questionnaire responses was performed in IBM^®^ SPSS^®^ Software (version 21, Chicago, IL, United States). The significance level of all statistical test was set to *α* = 0.05.

## Results

We did not find a significant effect of adding haptic disturbance (HD) during training in the error reduction from baseline to mid-training retention. As discussed above, using backward elimination and model comparison analysis with the AIC, the HD factor was eliminated from the LME model (Eq. 8).

### Performance During Training With Different Training Strategies

We used a LME model (Eq. 8) with five levels in time factor (baseline, training 1.1, training 1.2, training 2.1, and training 2.2), three levels in training group (Control, HEA, and VEA) and their interaction in order to analyze the participants’ performance during training. We selected the Control group as the reference level for group factor and baseline for the time factor in the contrast analysis.

**FIGURE 5:**
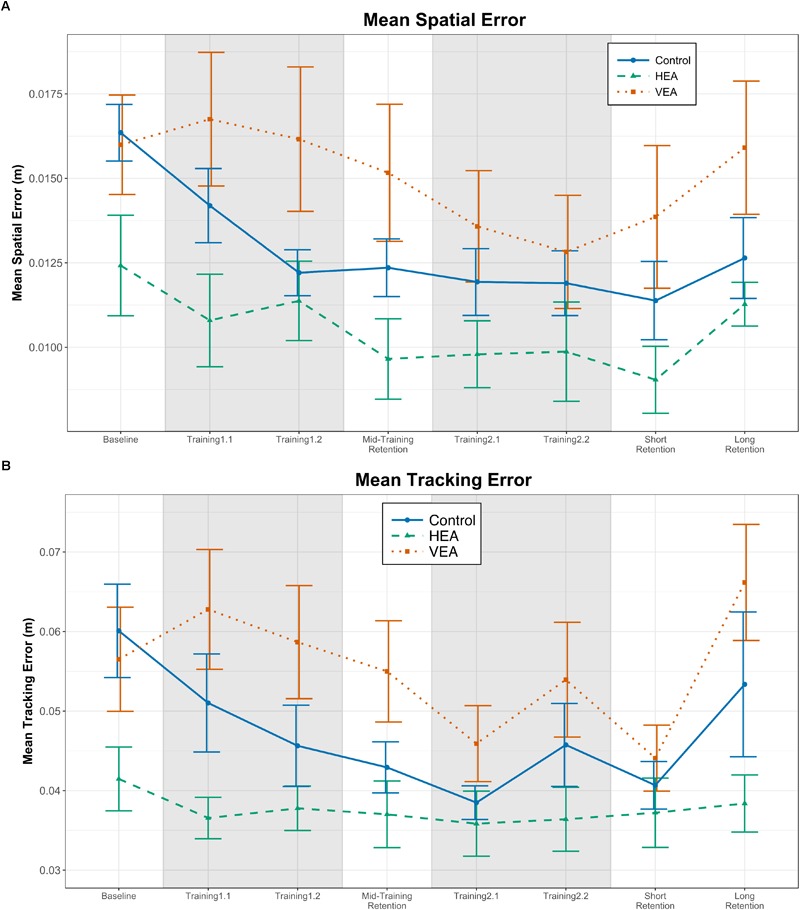
Effect of the training strategies on performance during training (shadowed trials) and retention tests. **(A)** Mean spatial error. **(B)** Tracking errors. Error bars: ±1 SE.

When employing spatial error as dependent variable, we found a significant time effect [Figure 5A, *F*(4,104) = 21.002, *p* < 0.001] and interaction between time and training group [Figure 5A, *F*(8,104) = 3.192, *p* = 0.003]. In particular, participants in the VEA group increased the spatial error systematically more than the Control group from baseline to training 1.1 (Table 2, *p* = 0.007), and training 1.2 (Table 2, *p* < 0.001). Similarly, the VEA increased the spatial error systematically more than the HEA group from baseline to training 1.1 [Figure 5A, β = 0.002, *t*(104) = 2.175, *p* = 0.031]. We also found that participants in the Control group reduced the error from baseline to training 1.2 in a significantly greater amount than the HEA group (Table 2, *p* = 0.006). However, we also found differences between training groups in the spatial error during baseline [Figure 5A, ANOVA: *F*(2,26) = 2.74, *p* = 0.083]. *Post hoc* comparisons revealed that participants in the HEA group performed systematically better than the Control group during baseline, although the difference did not reach significance (*p* = 0.102). Similar results were observed within the tracking error (see Figure 5B and Supplementary Table A1).

**Table 2:** Results from the linear mixed-effects model with training blocks as time factors (Baseline, Training 1.1, Training 1.2, Training 2.1, and Training 2.2) and spatial error as dependent variable.

	Estimate	*SE*	95% CI	*p*-value
Intercept	0.016	0.001	0.014, 0.019	<0.001***
HEA	-0.004	0.002	-0.008, 0.001	0.058^⋅^
VEA	-0.001	0.002	-0.004, 0.003	0.855
Training 1.1	-0.002	0.001	-0.004, -0.001	0.005**
Training 1.2	-0.004	0.001	-0.006, -0.003	<0.001***
Training 2.1	-0.004	0.001	-0.006, -0.003	<0.001***
Training 2.2	-0.005	0.001	-0.006, -0.003	<0.001***
HEA × Training 1.1	0.001	0.001	-0.001, 0.003	0.626
VEA × Training 1.1	0.003	0.001	0.001, 0.005	0.007**
HEA × Training 1.2	0.003	0.001	0.001, 0.005	0.006**
VEA × Training 1.2	0.004	0.001	0.002, 0.007	<0.001***
HEA × Training 2.1	0.002	0.001	-0.001, 0.004	0.105
VEA × Training 2.1	0.002	0.001	-0.001, 0.004	0.063^⋅^
HEA × Training 2.2	0.002	0.001	-0.001, 0.004	0.085^⋅^
VEA × Training 2.2	0.001	0.001	-0.001, 0.003	0.23

*SE, standard error; CI, confidence interval using parametric bootstrapping. Reference level for group factor is Control and for time factor is Baseline. ^∗∗∗^*p* ≤ 0.001, ^∗∗^*p* ≤ 0.01, ^∗^*p* ≤ 0.05, ^⋅^*p* ≤ 0.1.*

Results from the two-way ANOVA SPM analysis with the continuous tracking error as dependent variable confirm these observations. We found a significant time effect, indicated by two supra-threshold clusters in the test statistic trajectory (SPM{*F*}, at time frames 76–133 and 141–184 in the gait cycle) that exceeded the critical threshold of *F*^∗^ = 4.131 with *p* < 0.001. We also found a significant interaction between time and training group (time frames 105–159, *F*^∗^ = 3.090, *p* < 0.001). Differences across groups were found at the expected start of the swing phase (around the 100–150 time frames of the gait cycle, see Supplementary Figure A1). We note that usually there is a delay between the reference and actual ankle positions. Therefore, the differences noted around these time frames suggest that participants changed the timing of transitions between gait phases.

We found a significant main effect of adding haptic disturbance on top of the other training strategies in the variability of the spatial error during the first training block (LME with training groups [Control, HEA, and VEA], haptic disturbance groups [HD1, HD2] and their interaction as fixed effects; Supplementary Figure A2, training 1.2: *F*(1,23) = 6.928, *p* = 0.015). In particular, participants trained with visual or haptic error augmentation showed larger variability when haptic disturbance was added during training 1.2 compared to participants without haptic disturbance [VEA: β = -0.009, *t*(23) = -2.637, *p* = 0.015; HEA: β = -0.0067, *t*(23) = -1.842, *p* = 0.078]. During the second training block, the haptic disturbance was removed in the HD1 group and was added on top of the HD2 group (i.e., to the participants who were not trained with haptic disturbance during the first training block). We observed again that the variability was larger in participants trained with haptic disturbance on top of their main strategy [Supplementary Figure A2, training 2.1: *F*(1,23) = 6.554, *p* = 0.018]. In particular, adding haptic disturbance on top of the Control group significantly increased the variability during the second training block [training 2.1: β = 0.008, *t*(23) = 3.072, *p* = 0.005; training 2.2: β = 0.008, *t*(23) = 2.799, *p* = 0.01]. The differences in spatial variability between participants trained with and without haptic disturbance did not completely faded at short term retention [Supplementary Figure A2, *F*(1,23) = 5.34, *p* = 0.03].

### Effect of the Training Strategies on Motor Adaptation and Learning

Motor adaptation was evaluated using a LME model with the training groups (Control, HEA and VEA), time (baseline, mid-training retention, and short-term retention) and their interaction as fixed effects (Eq. 8). We selected the Control group as the reference level for group factor and baseline for the time factor in the contrast analysis.

In general, participants improved their performance, as suggested by a significant main time effect on spatial error [Figure 5A, *F*(2,52) = 29.04, *p* < 0.001]. We also found that the interaction between training group and time almost reached significance [Figure 5A, *F*(4,52) = 2.204, *p* = 0.061]. In particular, participants trained with VEA increased the spatial error from baseline to mid-training retention, while participants in the Control group reduced the error (Table 3, *p* = 0.008). This difference was also significant at short-term retention (Table 3, *p* = 0.016). We found that participants trained with HEA reduced significantly the spatial error when the HEA torques were removed from training 1.2 to mid-training retention [Figure 5B, paired *t*-test: *t*(8) = 2.66, *p* = 0.029]. However, we did not find significant differences in the tracking error between the last training test of each block and the following retention test. Similar results were observed within the tracking error (see Figure 5B and Supplementary Table A2).

**Table 3:** Results from the linear mixed-effects model with retention blocks as time factors [baseline, mid-training retention (MTR), short-term retention (STR)] and spatial error as dependent variable.

	Estimate	*SE*	95% CI	*p*-value
Intercept	0.016	0.001	0.014, 0.019	<0.001***
HEA	-0.004	0.002	-0.008, 0.001	0.064^⋅^
VEA	-0.001	0.002	-0.004, 0.003	0.859
MTR	-0.004	0.001	-0.006, -0.002	<0.001***
STR	-0.005	0.001	-0.007, -0.003	<0.001***
HEA × MTR	0.001	0.001	-0.001, 0.004	0.297
VEA × MTR	0.003	0.001	0.001, 0.005	0.008**
HEA × STR	0.002	0.001	-0.001, 0.004	0.180
VEA × STR	0.003	0.001	0.001, 0.006	0.016*

*SE, standard error; CI, confidence interval using parametric bootstrapping. Reference level for group factor is Control and for time factor is Baseline. ^∗∗∗^p ≤ 0.001, ^∗∗^p ≤ 0.01, ^∗^p ≤ 0.05, ^⋅^p ≤ 0.1*.

**FIGURE 6:**
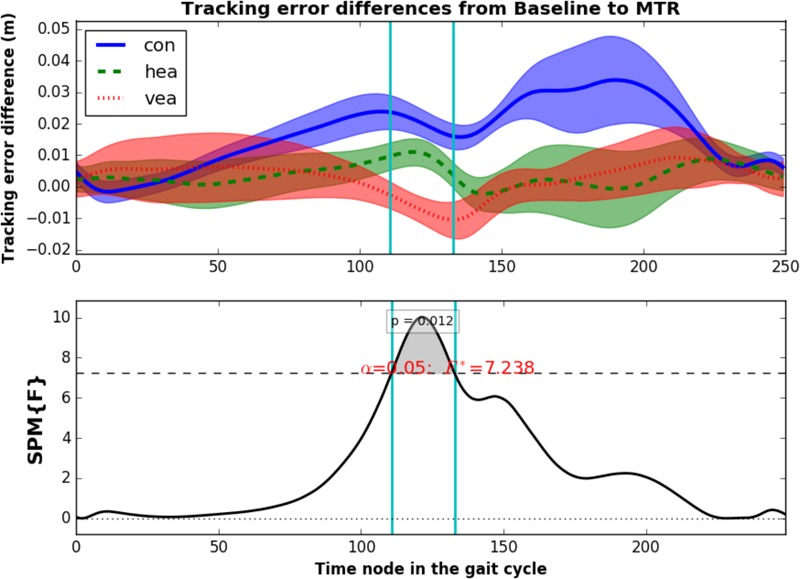
**(Up)** Trajectory of the tracking error reduction from baseline to mid-training retention. Positive values indicate that tracking error was reduced after training. Clouds represent the standard error. **(Bottom)** SPM{F} statistic for the one-way ANOVA with training groups as effect and tracking error reduction from baseline to mid-training retention as dependent variable. Vertical lines indicate starting and finishing time frames of the significant supra-threshold clusters.

Results from the two-way ANOVA SPM analysis of the continuous tracking error confirm these observations. We found a main effect of training group (time frames 96–120 exceeded the critical threshold of *F*^∗^ = 7.125 with *p* = 0.012), and time effect (two clusters exceeded the threshold of *F*^∗^ = 6.282, at 74–120 time frames with *p* < 0.001 and 231–249 time frames with *p* = 0.025). The interaction between training group and time almost reached significance (α = 0.1, time frames 139–149, *F*^∗^ = 3.905, *p* = 0.083). In particular, we found differences between training groups in the error reduction from baseline to mid-training retention (Figure 6, ANOVA, time frames 111–133, *F*^∗^ = 7.238, *p* = 0.0122). As observed during training, the differences between training groups were mostly found at the time frames around the first part of the swing phase, where the training strategies become active.

In general, participants reduced the spatial error from baseline to long-term retention (Figure 5A; LME with time [baseline and long-term retention], training group and their interaction as factors; main effect of time: *F*(1,26) = 5.827, *p* = 0.023). Not all training groups seemed to learn the target trajectory at the same extent (e.g., participants in the VEA did not reduced the spatial error at long-term). However, the interaction between training group and time did not reach significance [*F*(2,26) = 2.573, *p* = 0.096]. Participants showed a significant performance deterioration between the short- and long-term retention tests sessions (Figure 5A, LME with time [short- and long-term retention], training group and their interaction as factors; main effect of time: *F*(1,26) = 13.066, *p* = 0.001). On the other hand, we did not find a significant reduction of the tracking error from baseline to long-term retention. Participants showed a significant deterioration of their tracking performance between short- and long-term retention tests [Figure 5B, *F*(1,26) = 9.864, *p* = 0.004]. However, not all training groups seemed to worsen at the same extent (Figure 5B; time [short- and long-term retention] × group effect: *F*(2,26) = 2.46, *p* = 0.105). In particular, the tracking performance in the VEA and Control groups seemed to deteriorate more than the performance of participants trained with HEA (Figure 5B).

### Effect of the Training Strategies on Free Walking

We analyzed the effect of the different training strategies on gait asymmetry during the free walking tests performed in the middle of each training block (Figure 4) using LME models with training groups (Control, HEA and VEA), time (calibration, free walking 1 [FW1], and free walking 2 [FW2]) and their interaction as fixed effects (Eq. 8). The asymmetry of knee and hip joints between legs were employed as dependent variables.

**FIGURE 7:**
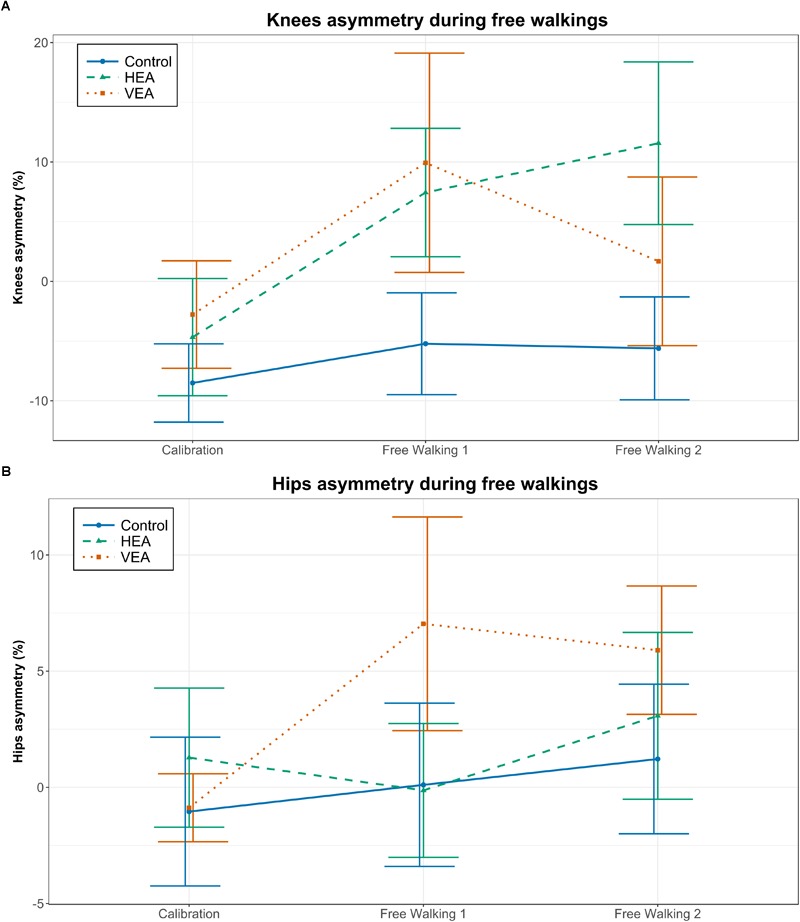
Effect of the training strategies on free walking. **(A)** Knee asymmetry. **(B)** Hip asymmetry. Error bars: ±1 SE.

We found a main time effect in the asymmetry between knees [Figure 7A, *F*(2,52) = 9.83, *p* < 0.001] and in the interaction of time and training group [Figure 7A, *F*(4,52) = 2.56, *p* = 0.049]. In particular, participants trained with HEA increased their knee asymmetry in a greater amount than participants in the Control group from calibration to the second free walking test (Table 4, *p* = 0.021) and participants who trained with VEA [β = -0.118, *t*(52) = -2.101, *p* = 0.041]. We did not find interaction effects between time and training groups in the hip asymmetry (see Figure 7B and Supplementary Table A3).

**Table 4:** Results from the linear mixed-effects with free walking blocks as time factors [Calibration, free walking 1 (FW1), free walking 2 (FW2)] model of impact in knees asymmetry.

	Estimate	*SE*	95% CI	*p*-value
Intercept	-0.085	0.057	-0.210, 0.034	0.144
HEA	0.038	0.083	-0.113, 0.197	0.646
VEA	0.057	0.081	-0.107, 0.228	0.482
FW1	0.033	0.040	-0.033, 0.110	0.399
FW2	0.029	0.040	-0.046, 0.101	0.457
HEA × FW1	0.088	0.056	-0.028, 0.197	0.122
VEA × FW1	0.094	0.055	-0.015, 0.199	0.091^⋅^
HEA × FW2	0.134	0.056	0.015, 0.238	0.021*
VEA × FW2	0.016	0.055	-0.074, 0.128	0.776

*SE, standard error; CI, confidence interval using parametric bootstrapping. Reference level for group factor is Control and for time factor is Calibration. ^∗^p ≤ 0.05, ^⋅^p ≤ 0.1.*

We further investigated the differences between the trained and non-trained joint trajectories using SPM. Two-way ANOVA with time effect (Calibration, free walking 1 and free walking 2) as repeated measures factor and training group as fixed effect (Control, HEA, and VEA) with knee trajectory differences between legs as the dependent variable showed significance on the time effect (at time frames 170–242, *F*^∗^ = 5.957, *p* < 0.001), indicating a significant increase of asymmetry between the knees during the free walking tests. The observed differences in the SPM plots occur mainly in the areas of maximum flexion (around the 170–210 time frames of the gait cycle, Supplementary Figure A1).

**FIGURE 8:**
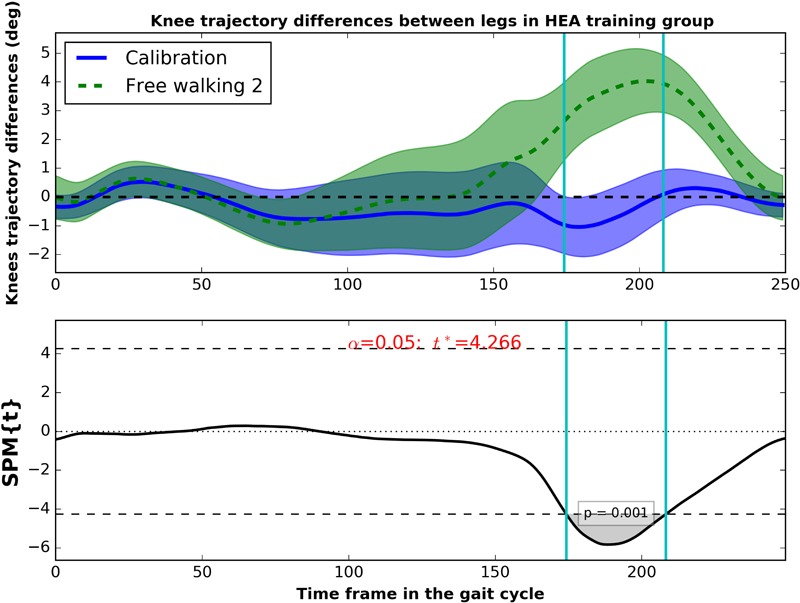
**(Up)** Mean trajectory of the knee angle differences during Calibration (solid blue) and free walking 2 (dashed green) from the HEA group. Clouds represent the standard error. **(Bottom)** SPM{t} statistic for the paired *t*-test (knee differences between Calibration and free walking 2) in the HEA group. Vertical cyan lines indicate starting and finishing points of the supra-threshold cluster in the test.

We performed a one-way ANOVA to evaluate the change from calibration to free walking 2 in the differences between knee trajectories (i.e., [θ_knee trained_ - θ_knee untrained_]_Calibration_ - [θ_knee trained_ - θ_knee untrained_]_FW2_). Although not significant, we observed that during the region of maximum knee flexion (170–210 time frames) participants trained with HEA showed a higher asymmetry (more negative values in knee trajectory differences) compared to the other training groups. In fact, only subjects trained with HEA significantly increased the knee asymmetry between calibration and the second free walking test (Figure 8, paired *t*-test, supra-threshold cluster at time frames 174–208 exceeding critical threshold of *t*^∗^ = -4.266 with *p* = 0.001). Positive values in this region indicate higher flexion on the trained knee compared to the untrained knee.

### Effect of Training Strategies on Motivation

**FIGURE 9:**
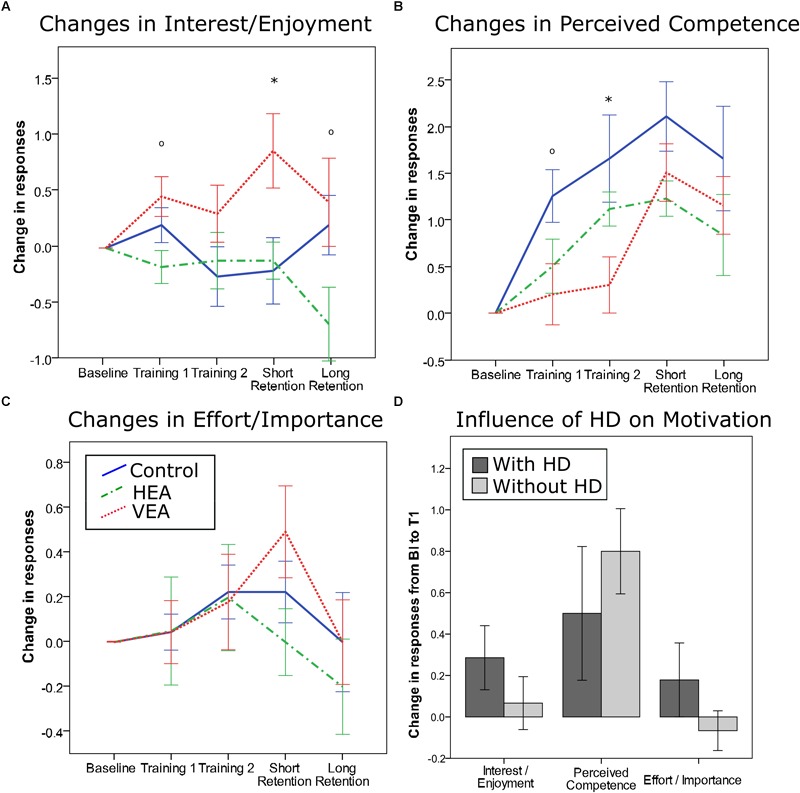
Effect of the training strategy on changes in responses to IMI subscales statements from baseline to each training block and after short and long retention tests. **(A)** Changes in responses assessing interest/enjoyment. **(B)** Changes in perceived competence. **(C)** Changes in effort/importance. **(D)** Changes in responses in each IMI subscale from baseline to first training session, for participants trained with haptic disturbance (HD) (dark gray) and without disturbance (light gray). ^∗^*p* < 0.05, °*p* < 0.1. Error bars: ±1 SE.

We found a significant main effect of training strategy on several subscales of the intrinsic motivation inventory. We found an almost significant effect in interest/enjoyment increase during training (Figure 9A, *p* = 0.058), a significant effect after short retention (Figure 9A, *p* = 0.042), and a one-side significant effect at long term (*p* = 0.090). In particular, participants in the VEA group reported a higher interest/enjoyment increase than participants in the HEA group (training: *p* = 0.028; short retention: *p* = 0.028), and participants in the Control group (retention: *p* = 0.043). We also found a significant effect of training strategy in the perceived competence during training (Figure 9B, *p* = 0.039). In particular, participants trained with VEA reported a lower perceived competence level compared to participants trained with HEA (*p* = 0.035) and the Control group (*p* = 0.043). We did not find a significant effect of the training strategy on effort/importance (Figure 9C).

Participants, who trained with haptic disturbance showed no significant changes in scores compared to those trained without disturbance in any of the IMI subscales (Figure 9D). However, when analyzing the effect of adding haptic disturbance only in participants trained with VEA and HEA, we found that participants trained with error amplification alone increased their perceived competence in a significant greater amount than participants who trained with error augmenting strategies plus haptic disturbance (Supplementary Figure A3, *p* = 0.017). This significant difference was not visible after the second training block.

## Discussion

### Training With Visual Error Amplification Hampered Performance and Motivation During Training

Based on the idea that errors are fundamental signals that drive motor adaptation (Emken and Reinkensmeyer, 2005), we expected better performance during training, since visually amplified movement errors would increase the detection and correction of small errors (Wei et al., 2005). However, training with visual error amplification was especially challenging, as suggested by the larger movement errors observed during the first training block, compared to the Control and haptic error amplification groups. This is corroborated by the limited reported perceived competence by the visual amplification group, contrary to the increased perceived competence reported by participants in the other training groups.

A possible explanation for this unexpected performance degradation might be originated in the value of the visual amplifying gain. Although we selected a relatively small gain (α_amp_ = 0.2), maybe the gain was still too large for participants to correctly interpret their performance loss during training. Previous experiments showed that doubling the visual errors (i.e., α_amp_ = 1), during training a reaching task resulted in faster adaptation (Patton et al., 2013) and better motor learning (Celik et al., 2009). It has been recently suggested that in order to accelerate learning of a point-to-point reaching task with visuomotor rotation, the gain should be 0.92, and for the fastest learning in combination with the best post-training performance, the gain should be decreased from 0.92 to 0 throughout training (Parmar and Patton, 2015). However, previous research aimed to amplify spatial errors in reaching point to point tasks –i.e., discrete simple movements– while in the current experiment, participants were requested to perform a continuous tracking task with their ankle –i.e., a rhythmic continuous task– while we amplified tracking errors (i.e., spatio-temporal errors). Therefore, it cannot be ensured that visual amplification gains that successfully work in learning simple point-to-point reaching task would also help learning more complex tasks. In fact, in a recent experiment, gains bigger than 0.4 confused participants when tracking a complex rowing stroke –i.e., a rhythmic continuous movement (Basalp et al., 2016). The gain of 0.2 was probably too large in this specific complex task, especially during the first training block. A possible solution might be to employ an adaptive visual amplification gain that is augmented based on participants’ ongoing errors (Rauter et al., 2011).

Training with haptic error amplification, on the other hand, did not result in poor performance during training, probably because large errors were limited with haptic guidance. A well-known potential limitation of haptic strategies is that participants might rely on the haptic guidance during training, and therefore, might fail to actively perform the task by themselves (Reinkensmeyer et al., 2009). However, we did not find a performance degradation when the haptic error amplification strategy was removed during the retention tests. In fact, participants performed significantly better when the haptic error amplification was removed during the mid-training retention test, suggesting that small tracking errors were, indeed, amplified.

### Training With Visual Error Amplification Hampered Motor Adaptation of the Locomotor Task

In general, all participants improved their performance already after the first training block. However, when comparing between training strategies, we found that participants trained with visual error amplification reduced their errors after the first training block (mid-training retention) significantly less than participants in the Control group. This difference was maintained after the second training block (at short-term retention). The motor adaptation limitation observed in the visual error amplification group could be explained by the poor performance and motivation observed during training. Probably, participants did not benefit from the large errors created during training because they failed to understand the reason behind their performance loss. Based on these results, it is essential to reexamine the simplistic interpretation of error-based theories in motor learning, i.e., that larger errors drive faster adaptation. It is crucial to evaluate with greater detail under what task conditions, and for what kind of errors, visual error amplification may benefit motor learning. An optimal framework might be, similarly to the haptic error modulating controller here presented, to visually amplify medium-sized errors that might be optimal for learning, while reducing large errors that can be frustrating to the participants. We note that in the visual error amplification strategy presented here we limited the amount of error amplified (to a maximum of 15°), but no error reduction was implemented.

Training with haptic error amplification, on the other hand, did not hamper the adaptation process. We found a smaller tracking error reduction after the second training block (at short-term retention), compared to the Control group. However, this difference might be originated in the initially better performance observed in the haptic error amplification group during baseline. Probably their potential to further improve was limited (ceiling effect). In previous studies we found that the specific characteristics of the motor task to be learned might play an important role on the effectiveness of robotic training (Marchal-Crespo et al., 2015b). In particular, we found that training with haptic guidance seemed to hamper learning of continuous rhythmic tasks (Marchal-Crespo et al., 2015a). Although haptic guidance was applied during training of the walking task presented here (i.e., a continuous rhythmic task) when errors were larger than a preselected threshold, this did not hamper the learning of the continuous rhythmic task. Probably, the addition of the haptic error amplification when the errors were sufficiently small prevented participants to rely on the guidance and promoted motor adaptation.

The statistical parametric mapping analysis revealed that the differences across training groups were mainly found at the time frames around the first part of the swing phase, where the training strategies become active. Note that increasing the joints’ ROM to create the reference ankle trajectory resulted in longer and higher steps along with longer swing phases. Therefore, the differences noted around the beginning of the swing phase suggest that participants in the different training groups adapted differently how to time the transition between gait phases of the reference trajectory. This is in line with several studies that have suggested that haptic demonstration of optimal timing, rather than movement magnitude, may facilitate skill transfer (Heuer and Lüttgen, 2015; Milot et al., 2018).

Nevertheless, in a recent experiment we found that the most effective robotic training condition depended on the characteristics of the task to be learned. We employed a similar haptic error amplification strategy in a 7 DoF robotic exoskeleton for upper limb rehabilitation (Marchal-Crespo et al., 2017a). In an experiment with thirty healthy participants, we evaluated the effectiveness of three error-modulating training strategies -no guidance, haptic error amplification and haptic guidance- on self-reported motivation and learning of continuous and discrete tasks. We found that training with haptic error amplification seemed to be especially suitable to enhance learning of discrete tasks, but did not result in better learning of a continuous task. This is in line with the results reported here. Participants probably benefited from the haptic error amplification provided during the transition between stance and swing phase to better time the gait cycle phases (i.e., time discrete task), but the benefit was limited in the overall continuous task (tracking a desired trajectory presented on a visual display with the ankle is a continuous task). We speculated that the lack of improvement when training continuous tasks with haptic error amplification might be linked to the specificity-of-learning hypothesis, which states that learning is most effective when training is performed involving the most crucial sensory information source needed to perform the motor task in retention tests. In both experiments, concurrent visual information was crucial in order to perform the continuous task, and therefore, maybe other sources of sensory information -for example proprioception- were neglected (Proteau, 2005).

In general, participants learned to perform the asymmetric desired trajectory (i.e., they reduced the spatial errors at long-term retention). However, not all training groups seemed to learn the target trajectory at the same extent. In fact, participants in the visual error amplification did not reduced the spatial error at long-term. However, the interaction between training group and error reduction from baseline to long-term retention did not reach significance. In general, participants did not learn how to precisely track the desired ankle trajectory (the tracking error reduction al long-term retention was non-significant). We observed tracking performance differences between short and long- term retention tests. Participants trained with haptic error amplification seemed to retain the improved tracking performance at long term, while participants trained with visual error amplification showed a significant tracking performance deterioration at long term. However, caution must be taken when driving conclusions from long-term retention results. Participants, in general, did not reduce the tracking errors at long-term retention. These lack of lasting effects on tracking error at long retention might be due to the too long time between experimental days -retention tests are usually performed after only 1–2 days (Heuer and Lüttgen, 2014; Duarte and Reinkensmeyer, 2015)- and due to the relative short training time (four training intervals of 2 min each).

As discussed above, the selection of the visual gain might play a crucial role on the effectiveness of visual error amplification in motor learning (Parmar and Patton, 2015; Basalp et al., 2016). Thus, we cannot categorically conclude that visual error amplification hampers motor adaptation and learning. Other visual amplifying gains (e.g., gains that are depended on the participants’ ongoing error) should be systematically evaluated in order to define the values that might improve adaptation and learning of a complex locomotor task.

### Training With Haptic Error Amplification Enhanced Transfer of the Practiced Asymmetric Gait Pattern to Free Walking

Training with haptic error amplification facilitated transfer of the practiced asymmetric gait pattern, as suggested by the more prominent gait asymmetry observed during the free walking tests, compared to the other training groups. Participants trained with haptic error amplification significantly increased their knee asymmetry by 16% (just below the 20% ROM increase employed to create the new gait pattern), even if they were instructed to walk naturally. In particular, we observed that during the region of maximum knee flexion, participants trained with haptic error amplification showed a higher asymmetry compared to the other training groups. In fact, only participants trained with haptic error amplification showed a significant change in asymmetry after training. This difference was more evident during the second training block, when participants already trained the task for 6 min with haptic error amplification. This finding is of special relevance in the field of robotic gait training. The aim of gait rehabilitation is that the gains observed during training are transferred to overground walking when the haptic and/or visual feedback employed during training is removed. Interestingly, training with only visual feedback (Control), and visual error amplification did not result in transfer of the practiced gait pattern, suggesting that the addition of robotic torques on top of the visual feedback had a positive effect on transfer.

This is in line with previous studies that found that robotic gait training with resistive forces applied during the swing phase results in improvements in walking function in post-stroke (Savin et al., 2014; Yen et al., 2015) and spinal cord injured subjects (Houldin et al., 2011; Yen et al., 2012). These walking improvements have been associated to the after-effects that appear when external forces –to which subjects have already adapted– are suddenly removed (Reisman et al., 2013). Furthermore, exposure to resistive forces may enhance muscle activation (Marchal-Crespo et al., 2014b). An additional explanation for the outperformance of the haptic error amplification strategy is that by adding forces on top of the experienced concurrent visual feedback, participants could benefit from more sensory inputs and improve motor adaptation (Wei and Patton, 2004). Some studies have suggested that multimodal feedback (i.e., the simultaneous addition of several sensory channels, such as haptic, auditory, and visual) enhances perception and action (Carson and Kelso, 2004; Seitz and Dinse, 2007), and may enhance learning of specially complex tasks (Sigrist et al., 2013, 2015).

### The Addition of Haptic Disturbance Increased Movement Variability During Training, but Had No Effect on Motor Adaptation

We found that, as expected, adding haptic disturbance increased the movement variability during training, especially in participants in the visual and haptic error amplification groups. However, the increased variability did not have a significant effect on motor adaptation. This is contrary to our hypothesis and to previous research that found a positive effect on adaptation when training with random feedforward torques (Lee and Choi, 2010; Marchal-Crespo et al., 2014b, 2017b). A possible rationale for this inconsistency is the relative short training duration under haptic disturbance (only two training intervals of 2 min each). A longer training duration with the addition of haptic disturbance might have resulted in different learning outcomes when compared to training without haptic disturbance.

Another rationale is that, in our experiment, haptic disturbance was added on top of the other training strategies that further augmented errors. Therefore, the effect of the haptic disturbance was augmented when applied on top of the error amplification strategies, independently whether the augmentation was done visually or haptically. This explanation is supported by the motivation results. When taking all participants together, we did not find a significant effect of adding haptic disturbance in any motivation subscale. However, we did find that participants trained with haptic disturbance on top of visual and haptic error amplification during the first training block (HD1 group) exhibited larger movement variability, compared to participants in the error amplification groups without haptic disturbance. Participants in the Control group, however, did not show larger variability when the disturbances were added during the first training block. The contrary effect was observed in the second training block: when haptic disturbance was added during the second training block (HD2 group), only participants in the Control group exhibited larger variability. Therefore, the way and order in which the haptic disturbance was added on top of the other training strategies had an impact on the error variability. Whether this has also an effect on motor learning needs further investigation in future work.

A decrease of perceived competence during training with haptic disturbance was also observed in a previous study (Marchal-Crespo et al., 2017a). Adding randomly varying disturbance torques during training complex 3D arm movements hampered learning and resulted in a decrease of feeling of competence when the haptic disturbance was applied. We hypothesized that this decrease in self-perceived competence probably reduced participants’ motivation to perform the task, and therefore, limited motor learning (McAuley et al., 1989). This is in line with a recent study which found that haptically amplifying errors reduced participants’ motivation and did not improve learning of a golf putting task (Duarte and Reinkensmeyer, 2015). Therefore, the positive effect of adding haptic disturbance to increase variability during training might have been limited by the negative effects of a decrease in perceived competence, especially in the groups trained with error augmentation. However, further experiments are needed to further evaluate the effect of different forms of haptic disturbance (e.g., different frequency and magnitude parameters) on motor learning.

### Training With Haptic Error Amplification Maintains Levels of Interest and Enjoyment and Leads to an Increase in Perceived Competence

As hypothesized, since the haptic error amplification strategy combined simultaneously haptic guidance and error amplification, it did not impact negatively on participants’ motivation, compared to the Control group. Participants trained with haptic error amplification maintained the level of interest/enjoyment during training and retention. Participants in the visual error amplification group, on the other hand, increased their interest/enjoyment during training in a greater amount that participants in the other training groups. This difference was more evident after the short-term retention test. At short-term retention, all participants performed the task without any guidance or disturbance from the robot, therefore the observed significant difference at retention might be related to the increase of perceived competence reported when the visual error amplification was removed. In fact, during training, participants in the visual error amplification group reported significantly lower values of perceived competence than participants in the Control and haptic error amplification groups. This difference, however, vanished once the visual error amplification was removed at retention tests.

Participants trained with the novel haptic error amplification strategy that combines haptic guidance and error amplification did not show significant differences in the evolution of the perceived competence with respect to the Control group. Participants probably benefited from the effect that error amplification had on keeping the interest and enjoyment during training, while the haptic guidance helped to increase the perceived competence as training progressed. Therefore, the novel designed haptic error amplification strategy kept the participants’ interest and enjoyment during training without negatively affecting their perceived competence.

### Experimental Design Limitations

The experimental design suffers from some limitations. First, the number of training blocks seemed to be insufficient to drive learning of some aspects of the motor task. Participants learned to perform the asymmetric desired trajectory (as suggested by a significant spatial error reduction at long-term retention) but did not learn how to precisely track the desired ankle movement at each time frame (the tracking error reduction al long-term retention was non-significant). The number of training blocks was decided after previous experiments that showed that performance of a similar locomotor task reached a plateau after five training intervals (Krishnan et al., 2013; Ranganathan et al., 2016). However, these previous experiments only accounted for a change in the performance, rather than learning effects (i.e., no changes in performance were tested at long term). Future research should include a larger number of training blocks during the first experimental day, or at different time points (e.g., after 1, 3, and 7 days) to evaluate whether learning of the tracking task (and differences between training groups) can also be observed at long-term retention.

Second, the effect of the haptic disturbance was augmented when applied on top of the error amplification strategies. Therefore, the analysis of the effect of haptic disturbance on motor adaptation is limited, as its effect on the participants trained with error augmenting strategies differs from that of participants in the control group. Finally, while haptic error amplification limited large errors while augmenting small-medium errors, visual error amplification augmented the errors, independently of their size (although we saturated the amplification at a certain error level). An interesting direction for future research is to perform further studies to evaluate a visual error amplification paradigm that visually amplifies medium-sized errors that might be optimal for learning, while reducing large errors that can be frustrating to the participants.

### Implications for Robot-Aided Gait Rehabilitation

During the last years, few studies have evaluated the use of resistive training strategies during robotic gait training. Robotic training with resistive forces applied during the swing phase resulted in improvements in walking function in individuals post-stroke (Savin et al., 2014; Yen et al., 2015) and spinal cord injured subjects (Houldin et al., 2011; Yen et al., 2012) compared to training with assistance. Similar outcomes have been observed in stroke patients when increasing participants’ walking asymmetry through a split-belt treadmill intervention (Reisman et al., 2013). Although training with resistive forces seems to improve motor function, training with these challenging strategies might also be associated with a long-term decrease on perceived competence and motivation (Duarte and Reinkensmeyer, 2015). Furthermore, applying external forces which reduce the patients’ performance during training might result in dangerous conditions, such as undesired stumbling.

Motor recovery is associated with brain plasticity induced by active training (Cramer et al., 2011). Similar cortical changes have been observed during the acquisition of new motor skills (Lotze et al., 2003). In fact, it is commonly accepted that recovery is a form of motor learning (or relearning) (Dietz and Ward, 2015). The novel haptic error amplification strategy presented in this paper, contrary to prior resistive training strategies, was developed based on well-established motor learning theories. The novel error modulating strategy limited dangerous and frustrating large error, while augmented smaller task-relevant errors. We found that training with this controller did not hamper adaptation and, in fact, resulted in good transfer of the practiced task to free walking. Furthermore, the haptic guidance limited performance errors during training, avoided participants to rely on the guidance and did not hamper the self-reported level of perceived competence, neither reduced the reported interest and enjoyment during training. Taking all this into account, we hypothesize that this novel haptic error amplification strategy might be a good framework to improve robotic gait training in neurological patients.

## Conclusion

We have shown that training with visual error amplification is specially challenging, as suggested by a performance degradation and decrease in the reported perceived competence during training. Training with visual error amplification also hampers motor learning of the locomotor task. Training with haptic error amplification facilitates transfer of the new asymmetric gait pattern during free walking, as suggested by a more prominent asymmetry between the legs after training. Adding haptic disturbance on top of the other training strategies increases the movement variability during training. However, increasing the variability during training does not improve motor adaptation, probably because the unforeseen random torques reduce the self-reported motivation level, especially in participants trained with visual and haptic error amplification. The differences observed between training strategies are predominantly localized during the first half of the swing phase.

The novel haptic error amplification strategy presented in this paper, which limits unsafe and frustrating large errors with haptic guidance while haptically augmenting small errors by means of error amplification, was developed considering well-established motor learning theories. Therefore, we hypothesize that the proposed haptic error amplification strategy might be a promising framework to improve robotic gait training in neurological patients. Further investigations with neurological patients are needed to corroborate this hypothesis.

## Author Contributions

LM-C, SM, and RR contributed to the experimental design and project supervision. LM-C and DO participated in the study design and data acquisition. LM-C and PT performed the data analysis and interpretation of the results. LM-C, PT, and RR prepared the manuscript. All authors read and approved the final manuscript.

## Conflict of Interest Statement

SM is employed by Hocoma. The remaining authors declare that the research was conducted in the absence of any commercial or financial relationships that could be construed as a potential conflict of interest.

## Footnotes

1 www.python.org
