# Supplementary material for: Haptic Error Modulation Outperforms Visual Error Amplification When Learning a Modified Gait Pattern
Source: Front Neurosci. 2019 Feb 19;13:61. doi: 10.3389/fnins.2019.00061 (PMC6390202; doi:10.3389/fnins.2019.00061)
Supplement: Supplementary file 1 [file Table_1.pdf]

Table A1. Results from the linear mixed-effects model with training blocks as time factors (Baseline, Training1.1, Training1.2, Training2.1, Training2.2) and tracking error as dependent variable

	**Estimate**	**SE**	**95% CI**	***p-value***
Intercept	0.060	0.005	0.048, 0.070	$< 0.001^{***}$
HEA	−0.019	0.008	-0.033, -0.003	0.019^{*}
VEA	−0.004	0.008	-0.017, 0.014	0.636
Training1.1	−0.009	0.005	-0.019, -0.001	$0.077^{\cdot}$
Training1.2	−0.014	0.005	-0.025, -0.004	0.005^{**}
Training2.1	−0.021	0.005	-0.031, -0.011	$< 0.001^{***}$
Training2.2	−0.014	0.005	-0.024, -0.004	0.006^{**}
HEA × Training1.1	0.004	0.007	-0.011, 0.018	0.575
VEA × Training1.1	0.015	0.007	0.001, 0.028	0.035^{*}
HEA × Training1.2	0.010	0.007	-0.004, 0.026	0.148
VEA × Training1.2	0.016	0.007	0.001, 0.030	0.023^{*}
HEA × Training2.1	0.011	0.007	0.001, 0.031	0.033^{*}
VEA × Training2.1	0.013	0.007	-0.004, 0.025	0.130
HEA × Training2.2	0.009	0.007	-0.005, 0.024	0.212
VEA × Training2.2	0.012	0.007	-0.003, 0.025	0.104

SE: standard error; CI: confidence interval using parametric bootstrapping. Reference level for group factor is Control and for time factor is Baseline. ***$p \leq 0.001$, ** $p \leq 0.01$, * $p \leq 0.05$, ·$p \leq 0.1$
