# Supplementary material for: Haptic Error Modulation Outperforms Visual Error Amplification When Learning a Modified Gait Pattern
Source: Front Neurosci. 2019 Feb 19;13:61. doi: 10.3389/fnins.2019.00061 (PMC6390202; doi:10.3389/fnins.2019.00061)
Supplement: Supplementary file 2 [file Table_2.pdf]

Table A2. Results from the linear mixed-effects model with retention blocks as time factors (baseline, mid-training retention [MTR] and short-term retention [STR]) and tracking error as dependent variable

	**Estimate**	**SE**	**95% CI**	***p-value***
Intercept	0.060	0.005	0.049, 0.067	$< 0.001^{***}$
HEA	−0.019	0.007	-0.029, -0.003	0.009^{**}
VEA	−0.004	0.007	-0.019, 0.005	0.598
MTR	−0.017	0.005	-0.029, -0.007	$< 0.001^{***}$
STR	−0.019	0.005	-0.029, -0.009	$< 0.001^{***}$
HEA × MTR	0.013	0.007	-0.001, 0.028	$0.080^{\cdot}$
VEA × MTR	0.015	0.007	0.007, 0.034	0.028^{*}
HEA × STR	0.015	0.007	0.001, 0.03	0.038^{*}
VEA × STR	0.007	0.007	-0.003, 0.026	0.319

SE: standard error; CI: confidence interval using parametric bootstrapping. Reference level for group factor is Control and for time factor is Baseline. ***$p \leq 0.001$, ** $p \leq 0.01$, * $p \leq 0.05$, $\cdot p \leq 0.1$
