# Supplementary material for: Haptic Error Modulation Outperforms Visual Error Amplification When Learning a Modified Gait Pattern
Source: Front Neurosci. 2019 Feb 19;13:61. doi: 10.3389/fnins.2019.00061 (PMC6390202; doi:10.3389/fnins.2019.00061)
Supplement: Supplementary file 3 [file Table_3.pdf]

Table A3. Results from the linear mixed-effects model with free walking as time factors (Calibration, FW1, FW2) of impact in hips asymmetry.

	**Estimate**	**SE**	**95% CI**	***p-value***
Intercept	−0.010	0.032	-0.078, 0.053	0.745
HEA	0.023	0.046	-0.072, 0.123	0.619
VEA	0.016	0.045	-0.072, 0.094	0.971
FW1	0.012	0.027	-0.042, 0.065	0.676
FW2	0.023	0.027	-0.034, 0.079	0.413
HEA × FW1	−0.026	0.040	-0.106, 0.060	0.523
VEA × FW1	0.068	0.039	-0.011, 0.137	0.087·
HEA × FW2	−0.005	0.040	-0.094, 0.074	0.908
VEA × FW2	0.045	0.039	-0.029, 0.124	0.249

SE: standard error; CI: confidence interval using parametric bootstrapping. Reference level for group factor is Control and for time factor is Calibration. * $p \leq 0.05$, $\cdot p \leq 0.1$.
