# Supplementary material for: Haptic Error Modulation Outperforms Visual Error Amplification When Learning a Modified Gait Pattern
Source: Front Neurosci. 2019 Feb 19;13:61. doi: 10.3389/fnins.2019.00061 (PMC6390202; doi:10.3389/fnins.2019.00061)
Supplement: Supplementary file 4 [file Image_1.pdf]

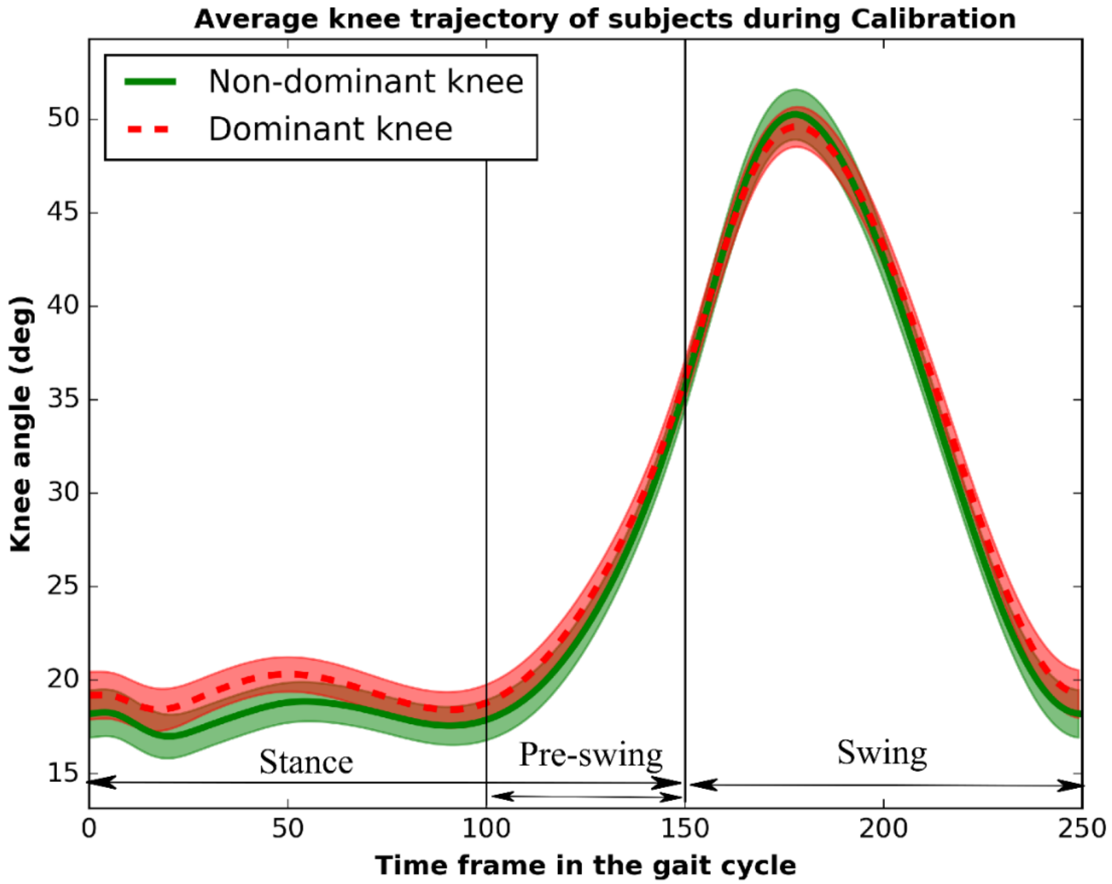

Figure A1: Average knee trajectory of dominant and non-dominant leg at the beginning of the experiment (Calibration) and average gait phases calculated as percentage of the whole gait cycle.
