# Supplementary material for: Haptic Error Modulation Outperforms Visual Error Amplification When Learning a Modified Gait Pattern
Source: Front Neurosci. 2019 Feb 19;13:61. doi: 10.3389/fnins.2019.00061 (PMC6390202; doi:10.3389/fnins.2019.00061)
Supplement: Supplementary file 5 [file Image_2.pdf]

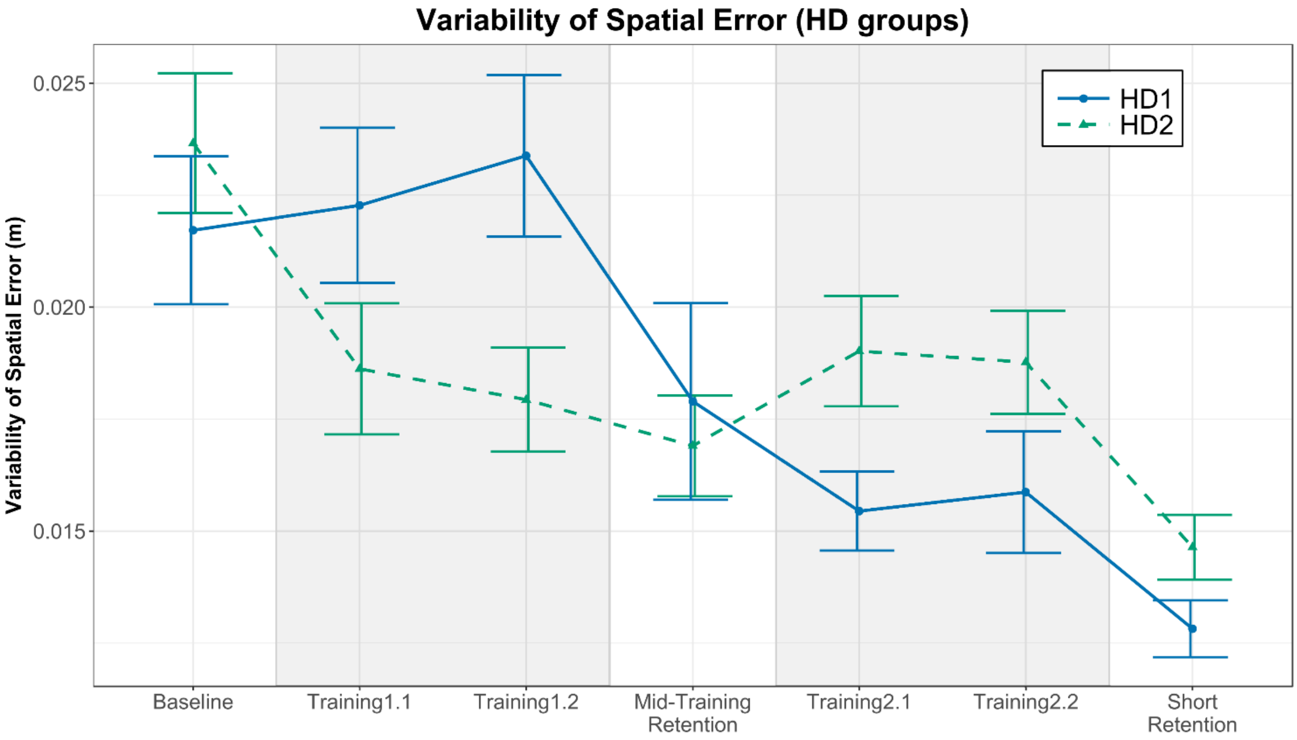

Figure A2: Effect of the addition of haptic disturbance (HD) on top of the other training strategies during the first training block (HD1) or second training block (HD2) on the variability of spatial errors during training (shadowed trials) and retention tests. Error bars: ±1 SE.
