# Supplementary material for: Haptic Error Modulation Outperforms Visual Error Amplification When Learning a Modified Gait Pattern
Source: Front Neurosci. 2019 Feb 19;13:61. doi: 10.3389/fnins.2019.00061 (PMC6390202; doi:10.3389/fnins.2019.00061)
Supplement: Supplementary file 6 [file Image_3.pdf]

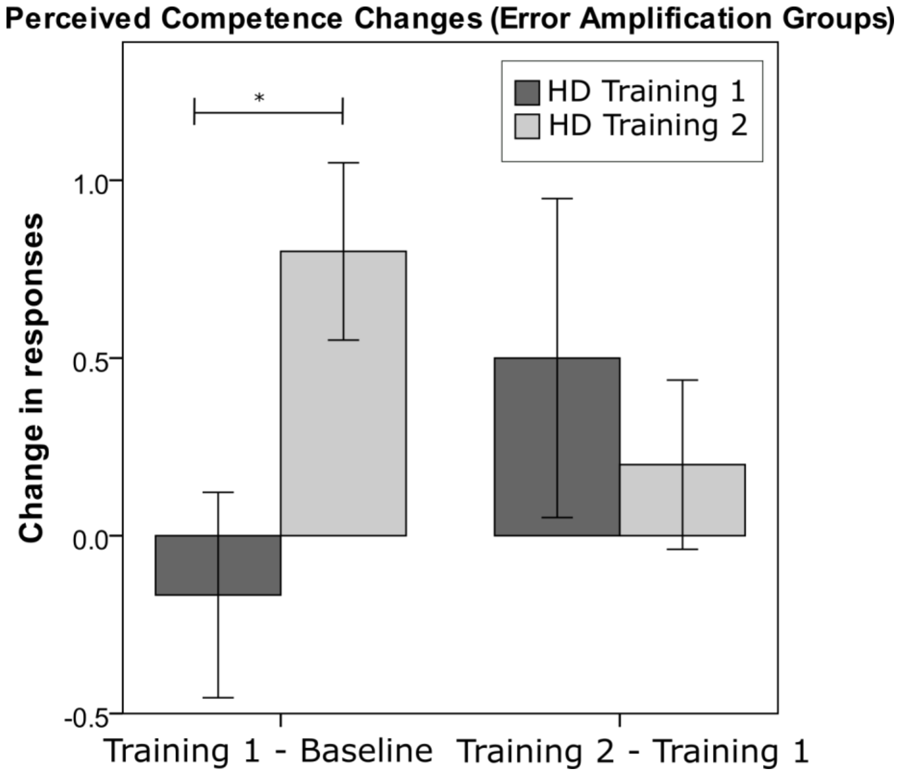

Figure A3: Changes on perceived competence after each training block for error amplification groups' subjects trained with haptic disturbance during training session 1 (dark grey) and with haptic disturbance during training session 2 (light grey). * $p < 0.05$, · $p < 0.1$. The asterisks show significant differences between training conditions for a given experimental phase. Error bars: ±1 SE.
